# Analytical and Experimental Research of Lubrication Load-Bearing Characteristics of Microtextured Meshing Interface

**DOI:** 10.3390/ma18040845

**Published:** 2025-02-14

**Authors:** Xigui Wang, Jiafu Ruan, Yongmei Wang, Weiqiang Zou

**Affiliations:** 1 School of Mechatronics and Automation, Huaqiao University, No. 668 Jimei Avenue, Jimei District, Xiamen 361021, China; lixledu1960@163.com (J.R.); zhaoxzhit@163.com (W.Z.); 2 School of Motorcar Engineering, Heilongjiang Institute of Technology, No. 999, Hongqidajie Road, Daowai District, Harbin 150036, China

**Keywords:** deep-sea gear, microtextured interface, meshing teeth surface, thermoelastic hydrodynamic lubrication, anti-scuffing load-bearing, experimental analysis

## Abstract

The excellent lubrication and load-bearing synergistic modulation of the meshing interface has been well recognized, as the microtextured tooth surface seems to be a punished area in deep-sea gear thermal elastohydrodynamic lubrication (TEHL). This is mainly because of the traditional perception of the anti-scuffing load-bearing capacity (ASLBC) and the similarity of the interfacial microelement configurations. Microtextured contact can be applied to the meshing interface to adjust the time-varying TEHL characteristics and enhance the meshing load-bearing performance. In this study, the analytical homogeneous equivalent micro-hydrodynamic contact multiscale parameters are determined, and the dispersed micro-flow real distribution area of the texturing interface is indicated, revealing the TEHL friction characteristics of the rolling–sliding line contact microelement, which is regarded as a bridge connecting the micro-dynamic pressure discrete contact friction behavior and the TEHL textured interface meshed-gear load-bearing. The contact model mentioned theoretically predicts the evolutionary time-varying characteristics of the micro-thermoelastic lubrication behavior of the textured contact interface under hydrodynamic conditions and demonstrates that the microtextured configuration parameters of the molecular scale meshing interface are the most influential structural parameters for the load-bearing problem of the homogeneous flow pressure film layer between the gear pair tooth surfaces, especially for deep-sea gear meshing load-bearing reliability under limited lubrication space.

## 1. Introduction

The interface texture is beneficial for forming the hydrodynamic fluid lubrication effect, which in turn improves the lubricating film load-bearing characteristics of the medium layer. In the actual meshing transient, the hydrodynamic fluid lubrication load-bearing characteristics and the mechanism of medium layer film formation at the interface during the actual meshing instant are still in need of in-depth analysis and research. Based on the preparation of the textured interface samples and the lubrication load-bearing experiments, the lubrication load-bearing capacity of the meshing medium film under TEHL conditions is evaluated for different parameters to investigate the lubrication-bearing characteristic law of the meshed interface and to explore the effect of the correlation mechanism between the microscopic morphology and the arrangement pattern of the textured microelements on the meshing interface, determining how to enhance the lubrication characteristic and the load-bearing performance. 

Considering the micro-textured multiscale element (MTME) feature to analyze the meshing interface micro-texture (MIMT) of the surface of the gear teeth is recognized as an effective technique to improve the meshing contact behavior and enhance the load-bearing capacity of the lubrication interface in a deep-sea gear transmission system (DSGTS) [1,2,3]. Under heavy load and low speed operating conditions, the medium dynamic pressure in the lubrication pattern of the meshing pair of the DSGTS plays a dominant role, which changes the surface contact of the meshing pair teeth and the interface lubrication state through the variable interface morphology of the microtextured configuration [4]. 

When the textured interface medium film is subjected to a certain load, the medium dynamic pressure effect generated by the designed microelements of the surface of the textured teeth significantly increases the medium film thickness between the lubricating interfaces of meshing pair, thereby effectively avoiding the non-indirect contact of the meshing pair micro-peak and reducing the coefficient of tooth surface friction [5]. Under the set operating conditions, the interface microtexturing parameters (configuration, scale, arrangement, etc.) become the critical factors affecting the hydrodynamic medium load-bearing performance of the gear surface, and efforts are made to seek the optimal combination of microtexturing parameters to enable the texturing interface to exhibit the best hydrodynamic load-bearing performance; this is the research goal in the field of microtexturing interfacing [6]. 

By introducing MTME meshing interfaces to induce geometric shape changes and establishing a lubrication performance simulation model using numerical analysis, the ASLBC of the meshing tooth surfaces can be enhanced at the same medium layer film thickness using the multi-element scale configuration [7]. The correlation of the load-bearing characteristics between the elastic medium hydrodynamic lubrication meshing tooth surfaces and the thermal behavior of the tribological contact problems is sensitive to parameter effects. These effects are widely present in the multi-dimensional range of the non-microscopic typical scale (typically, the nominal length of the meshing interface under thermoelastic medium hydrodynamic lubrication) to the non-macroscopic interlaminar scale [8]. The aforementioned scale corresponds to the preset topological configurations at the level of the meshing interface delineation parameters (geometry, dispersion effect, MTME structure, non-homogeneous distribution of medium layer film, etc.); using this scale, the MIMT elastic dynamic pressure medium lubrication model is constructed. The numerical analysis method is adopted, and the dimensionless average contact pressure is recognized as an indicator of the gear surface load-bearing performance, demonstrating that the interfacial microtexture configuration parameters influence the tooth surface homogeneous interface enriched lubrication (IEL) characteristics. The model is designed to obtain the ideal design parameters of the microtextured interface to optimize the homogeneous IEL characteristics, reduce the friction, and enhance the ASLBC of the meshing interface [9]. 

It has been found that parameters such as the interfacial texturing microelement scale, arrangement distribution, and morphology density play an important role in enhancing the thermoelastic ASLBC and improving the contact friction behavior of the tooth surface, and the effects should not be neglected [10,11,12]. By constructing a finite element analysis model, the influence of the laws of concave pits point geometric MTME on the tribological interface meshing load-bearing characteristics of the medium hydrodynamic lubrication is identified, and furthermore, the thermoelastic ASLBC is evaluated [13,14,15]. The microtextured interface morphologies (achieved by laser etching steel ring components to produce circular, longitudinal, and transverse texture appearances) are designed, and the frictional contact performance is studied. The results show that the depth gradient behavior of the composite MTME is governed by the relative sliding velocity between the interfaces [16,17,18]. To achieve the optimal contact characteristics of the meshing interface, the MTME configuration, or the chemically customized modification of the tooth surface, is designed in advance or used to simulate the line contact microtextured meshing interface during the rolling and sliding motion. Previous researchers have assumed that the lower the meshing interface roughness, the better the line contact characteristics will be. However, a thorough investigation has found that the meshing interface with geometric MTMEs achieve the desired homogeneous IEL characteristics, frictional contact behavior, and ASLBC.

**Figure 1:**
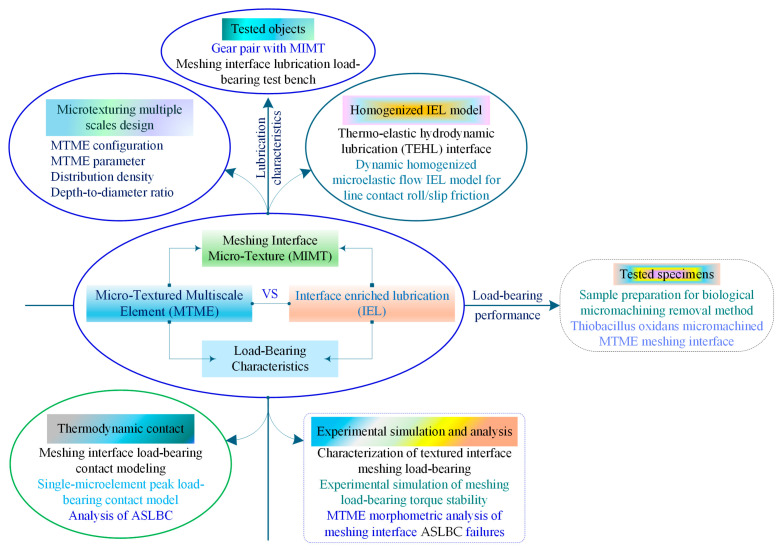
Organizational framework for the content of this study.

Figure 1 illustrates that the service performance and operational reliability of deep-sea gears are closely related to the morphology configuration design of the MTME of the meshing interface, lubrication enrichment (or IEL), and the ASLBC mechanism. However, the interrelated factors mentioned cover a complex mapping relationship, which presents an obstacle to the construction of a unified theoretical model that considers the homogeneous flow IEL characteristics and meshing load-bearing properties of the MTME interface in the thermoelastic steady state. The problem may be how to analyze the interface lubrication and load-bearing mechanisms characterized by the MTME parameters and to present a microelement contact meshing load-bearing model that considers the interfacial homogeneous flow-pressure lubrication.

**Figure 2:**
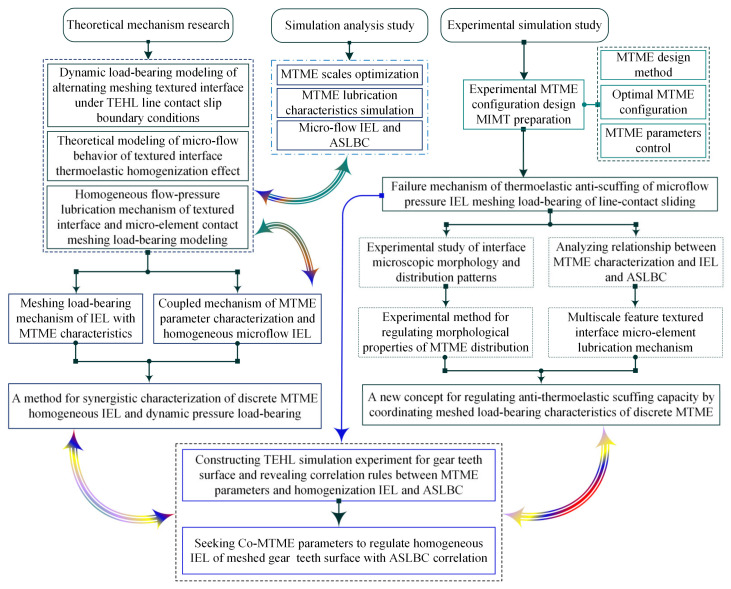
Technical lines of research for this thesis.

Figure 2 illustrates the technical lines of the research. A synergistic method of characterizing the discrete microelement homogeneous TEHL and the dynamic pressure load-bearing of MIMT is proposed, and TEHL-bearing simulation experiments using gear surfaces are constructed, revealing the correlation rules between MTME parameters and homogeneous IEL and ASLBC. Another goal of the study is to seek synergistic MTME parameters to regulate the homogeneous IEL in correlation with ASLBC, realizing a new conception of discrete microelement scale characteristics to control the anti-thermoelastic scuffing of the meshing load-bearing.

## 2. Interfacial Texturing Microelement Homogenization Thermoelastic Dynamic Pressure Contact Model

A microtextured effective contact dynamic lubrication method based on the principle of interface approximate homogenization is proposed to describe the problem of approximate homogeneous Reynolds fluid dynamic properties induced by the interface microtexture [19,20,21]. The MIMT configuration scale-multiplicity properties (microelement geometry, depth gradient, density pattern, arrangement, etc.) are determined, and the localized length time-varying properties of the roll–slip meshing of line-contact teeth surfaces due to the MTME units under viscous hysteretic fluidic TEHL are accounted for [22,23,24]. This study is an important step towards understanding the microscopic mechanisms underlying the meshing conditions of the texturing gear-pair line contact interface in DSGTS. An optimal solution strategy is proposed to analyze the dynamic-pressure contact characteristics of the textured meshing interface under TEHL conditions, and the MTME morphology is expected to be tuned to significantly improve the meshing load anti-thermoelastic scuffing performance. Based on the TEHL state, the MTME (such as the concave–convex peak element) is regarded as a micro-flow pressure medium lubrication bearing support, thereby generating an additional medium dynamic pressure contact force to effectively eliminate the direct sliding friction between the meshing interface and the rigid tooth surface. Under high-speed and heavy-load working conditions, the secondary lubrication effect of microtextured gears in DSGTS with additional dynamic pressure fluid film is identified as a key factor affecting the frictional meshing interface contact characteristics and tooth surface load-bearing capacity. Seeking the MIMT multiscale design and topology optimization methods to maximally improve the frictional contact characteristics of meshing pair and the tooth surface load-bearing performance in DSGTS has been regarded as the core direction of the MIMT technology. Although many scholars at home and abroad have already conducted a large number of studies by combining theoretical and experimental methods, achieving some encouraging results, there is still room for strengthening the systematic and universal correlation research, and there are significant differences between these results, which have not formed a complete framework of the MTME design theory thus far. The lubrication dynamic pressure load-bearing performance of different texturing interface microelement forms is also different. From the perspective of reducing friction and improving lubrication bearing properties, the discrete pothole MTME is superior to the continuous pothole MTME [25,26,27]. This paper aims to comprehensively investigate the tribological characteristics and meshing bearing performance of textured interfaces, with the goal of expanding the application fields of the universal principles of MIMT. It combines theory with experiments to systematically study the MTME parameters, geometric shapes, depth gradients, density morphologies, and arrangement patterns of MIMT. It analyzes the influence of microtexture on load-bearing performance under the TEHL state and reveals the mechanism by which texturing improves the meshing interface lubrication and load-bearing performance, providing guidance for the design of microtextured interfaces.

**Figure 3:**
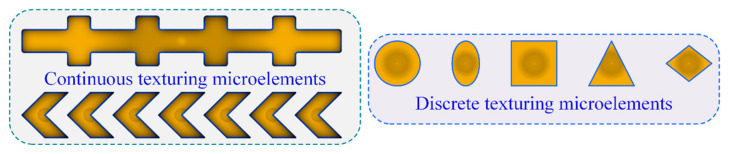
Discrete and continuous textures microelements.

**Figure 4:**
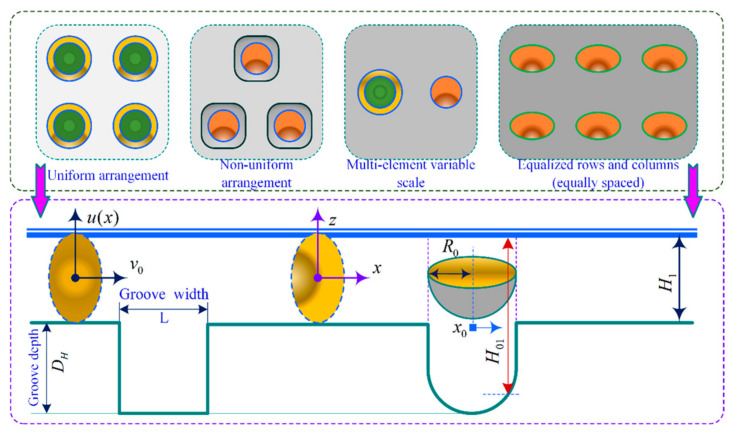
Multiscale characterization of textured microelement interfaces (homogeneous arrangement density and geometrical configuration parameters).

As shown in Figure 3, the MTME features (such as rectangular, circular, quasi-triangular, and honeycomb shapes) are extended to the discrete arrangement model, where the continuous arrangement MTMEs are presented in straight or curved arrays (such as parallel or crossed forms). The consideration of differences and similarities in multiscale features in the micro-interface configuration design will result in time-varying contact dynamic pressure distribution within the tooth surface meshing domain. The urgent technical problems that need to be solved for the texturing microelement interface include determining the relationship between the microscale features (length, width, and depth, as shown in Figure 4) and the edge critical dimensions (array and pitch) of the tooth surface contact profile.

The key parameters of the configuration design are determined in order to optimize the shape of the MTME and the scale correlation between the geometrical parameters, with a view to obtaining the interfacial meshing roll–slip contact friction to weaken the wear effect on the tooth surface and thus improve the lubrication performance. Thus, this method is applied toward the realization of the lubrication and load-bearing correlation of the tooth surface MIMT. Based on the thermoelastic effect of the hydrodynamic pressure lubrication state, the roll–slip line contact interface MTME and the geometry are preset to reveal the correlation between specific key parameters (e.g., microelement width and depth) affecting the friction behavior and load-bearing capacity under TEHL conditions. Setting the effective friction domain of the cylindrical pin simulation and the disc truncation surface TEHL to be regarded as the form of line contact at the meshing interface and identifying the Reynolds equation as the master equation for tooth surface fluid dynamic pressure lubrication of the meshing interface, its dimensionless form [28,29] is expressed as follows:

(1) $$\frac{\partial }{\partial x}\left(\xi \frac{\partial P}{\partial x}\right)-\left(\frac{\partial (H\rho_{m})}{\partial x}\right)=0$$

(2) $$\xi =\rho_{m}{\left(R_{x}H_{c}/a^{2}\right)}^{3}/(12\eta_{0}\mu_{m}R_{x}^{2}/P_{\max}a^{2})\eta_{m}$$

(3) $$U=\eta_{0}\mu_{m}/E_{1}R_{x}$$

(4) X=x/a

where $P_{\max}$ denotes the maximum value of the Hertzian contact pressure P. a is the half-width value in the contact domain of the meshing tooth surface. $H_{c}$ is the value of dynamic pressure fluid center layer film thickness H. $\rho_{m}$ is the fluid density of the Hertzian contact pressure P. $\mu_{m}$ denotes the average value of the coefficient of friction. $\eta_{0}$ is the dynamic pressure fluid viscosity. $\eta_{m}$ is the mean value of dynamic pressure fluid viscosity. $E_{1}$ expresses the equivalent value of the modulus of elasticity.

The dynamic pressure fluid film thickness and its average gap quantity are based on the same computational contact model, i.e., the behaviors of thermoelastic deformation, flow velocity, and roughness. Considering the region of fluid dynamic pressure lubrication, the interfacial contact pressure is controlled by the Reynolds equation; then, its expression [29] is as follows:

(5) $$U\frac{\partial (H_{c}\rho_{m})}{\partial x}+\frac{\partial (H_{c}\rho_{m})}{\partial t}=\frac{\partial }{\partial x}\left(\frac{\rho_{m}H_{c}^{3}}{12\eta_{m}}\frac{\partial P}{\partial x}\right)+\frac{\partial }{\partial y}\left(\frac{\rho_{m}H_{c}^{3}}{12\eta_{m}}\frac{\partial P}{\partial y}\right)$$

where the direction of the line contact roll–slip between the meshing interfaces coincides with its x-coordinate.

**Figure 5:**
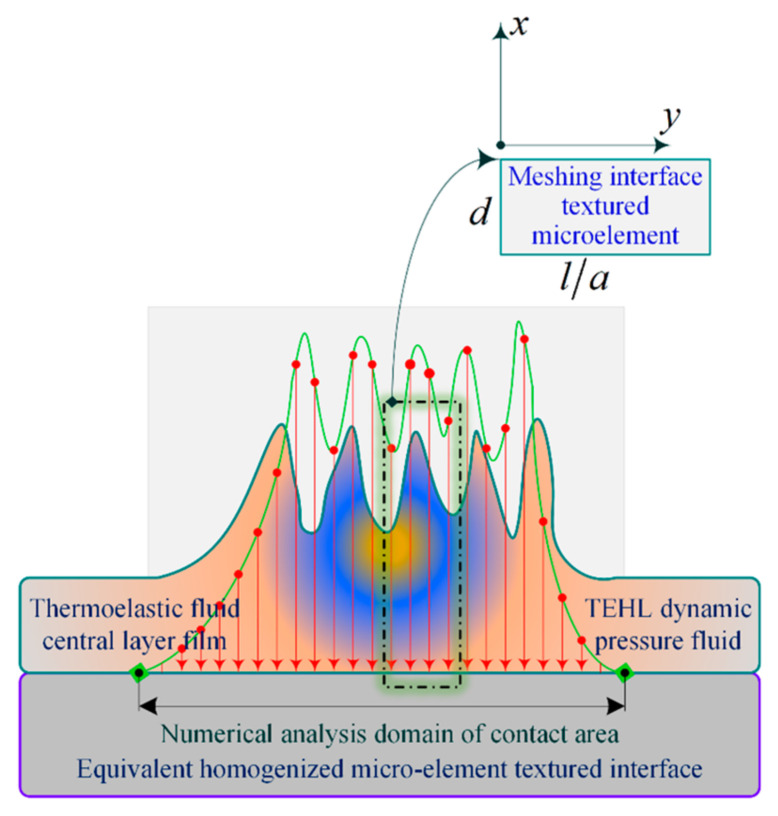
An equivalent homogenization of microelementary textured interfacial parts in resolved domains.

As shown in Figure 5, based on a non-finite-length linear contact cylinder rolling slip model, this is used to compute the equivalent homogenized microelementary textured interfacial parts (overlapping in the x direction) within the analytical domain of the numerical analytical domain of the TEHL dynamic fluid contact region. d is the depth of the meshing interface texturing microelement, and l/a is the aspect ratio of the individual texturing microelement. The approximation bias caused by the asymptotic classical assumption should not be ignored, and the multiscale characteristic differences of the textured microelement configurations make it difficult to obtain more accurate and optimal numerical solutions in the TEHL dynamic pressure line contact simulation analysis. Considering the periodicity and homogeneity of the multiscale features of the microelement of the texturing interface, a new concept of the formal decoupling method of the macroscopic and microscopic configuration scale is proposed to establish an equivalent homogenized microelement thermoelastic dynamic-pressure contact model in the meshing domain of the texturing interface, paying attention to the deformation behavior induced by the thermoelastic effect, as well as the load-bearing performance associated with the scale features of the microelement, which in turn extends the universality range of the asymptotically classical equivalent homogenized analytical method.

The dimensionless form of the thermoelastic dynamic pressure fluidic layer film thickness [30] is expressed as follows:

(6) $$H_{LFT}=\psi +H_{1}+x^{2}/2a^{2}$$

where ψ represents the elastic deformation considering the thermoelastic deformation effect and is described as follows:

(7) $$\psi =\left[\frac{(\left(v_{1}^{2}-1\right)/E_{1})+\left(v_{2}^{2}-1\right)/E_{2}+\cdot \cdot \cdot \left(v_{n}^{2}-1\right)/E_{n}}{\pi }\right]\int_{x_{a}}^{x_{a^{\prime }}}P(x)\ln{\left(\frac{x}{R_{0}}-\frac{x^{\prime }}{R_{0}}\right)}^{2}dx$$

Based on the previous research results, when the thermal expansion elastic stiffness coefficient of the meshing interface increases, the numerical-analytical convergence speed within the meshing domain of the contact area decreases, and is concluded. This implies that the interface thermal expansion elastic stiffness coefficient is not effective in suppressing the meshing impacts but only improves the dynamic pressure lubrication performance, wear friction behavior, and load-bearing capacity fluctuation of the line contact rolling–sliding meshing interface.

To seek an optimal scale characterization method to describe the conformational shape of the texturing microelements and the scale characterization quantity, the texturing microelements should contain at least the length-to-width ratio of the individual microelement concave–convex body, the parameter κ, and the minimum value of the dimensionless fluid layer film thickness $d_{s}$ between the meshing interfaces; then, the expression is: $\kappa =\frac{D_{h}}{2R_{0}}$, $d_{s}=\frac{H_{c}}{2R_{0}}$. Where the x geometric center is defined by a texturing microelement with radius $R_{0}$ and depth $D_{h}$, as shown in Figure 2 and Figure 3. In Equation (6), which further revises the contribution of the association between the texturing microelement and the fluidic layer film thickness of the meshing interface into Equation (8), expressed as [31]:

(8) $$\frac{H_{01}(x)}{H_{1}}=\left\{\begin{array}{l} \frac{\left(\frac{4\kappa^{2}-1}{4\kappa }\right)+\sqrt{\left(\frac{1+4\kappa^{2}}{2\kappa }\right)-x^{2}}}{2d_{s}}+1,\left(x\leq 1\right)\\ 1,\left(x>1\right) \end{array}\right.$$

The dynamic pressure fluid parameters (viscosity–density properties) between the meshing interfaces are varied by the effects of viscosity–temperature and viscosity–pressure. To correct the influence of the temperature–pressure function on the dynamic pressure fluid viscosity [32], the Roelands characterization Equation (9) is introduced, which is expressed as follows:

(9) $$\frac{\eta }{\eta_{0}}=\exp\left[-\left(\ln\eta_{0}+9.67\right)\left({\left(5.1\times 10^{-9}P+1\right)}^{0.68}-1\right)\right]$$

Using the Dowson and Higginson method to derive a correlation law between the density and pressure parameter indicators [33,34,35], we find the following:

(10) $$\frac{\rho }{\rho_{0}}=\left(1+\frac{6P}{10+17P}\right)$$

The equilibrium equation for the line contact meshing load-bearing problem based on a textured interface is expressed as the integral of the product of the contact area of the microtexturing unit, and its pressure over the entire meshing domain is equal to the total applied load [36,37,38], which is expressed in a dimensionless form as follows:

(11) $$\varpi =\int_{-\infty }^{+\infty }Pdx$$

where ϖ is a parameter that evaluates the meshing interface textured microelement load-bearing index.

**Figure 6:**
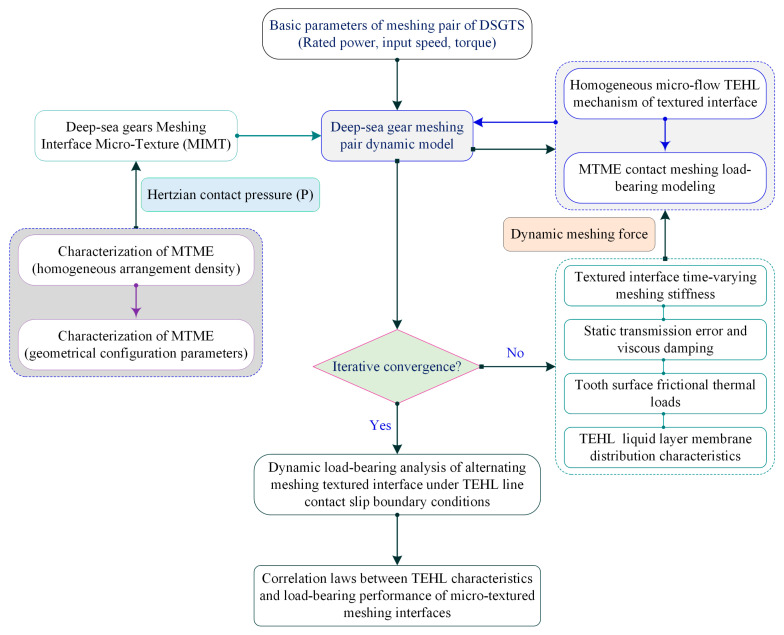
An algorithm flow chart of the texture multiscale microelementary lubrication model accounting for ASLBC capacity enhancement.

Figure 6 presents the numerical algorithm flow of the texture multiscale microelement lubrication model, considering the ASLBC capacity enhancement, thereby obtaining the expected simulation data and predicting the friction contact coefficient at different speeds. The simulation results show that when the interface slip speed increases, the thickness of the fluid layer film between the tooth surfaces also increases, and the friction contact pressure decreases. By comparing the time-varying interface speed of the homogenized texture equivalent microelement with the simulated friction coefficient, the simulation values of the friction coefficient at different slip speeds are obtained, and the optimal microtexture type with better uniformity is identified.

**Figure 7:**
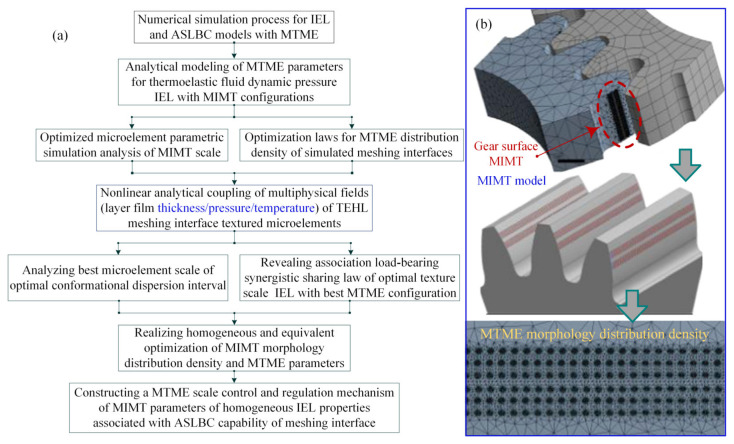
Illustration of microtextured gear pair meshing simulation: (**a**) analysis process flowchart; (**b**) MIMT model and MTME morphology scale distribution density.

The spur gear pair is considered to be non-flexibly supported. Table 1 illustrates the gear geometry parameters of the finite element model analysis. The microtextured spur gear pair meshing simulation process is shown in Figure 7. The numerical solution of the Reynolds equation is calculated based on the Newton–Raphson simulation method, which is faster than the direct solution of the conventional algorithm and offers superior convergence under high loads in real time at the line contact interface.

The numerical simulation analysis can be briefly described as follows: by using the finite difference method to regulate the texture microelement equation and perform discretization processing, the layout and arrangement characteristics of the texture microelement scale are established, the initial contact pressure at the meshing interface is determined, the thickness value of the dynamic pressure fluid film between the tooth surface interfaces is calculated, and the optimal value of the minimum fluid film thickness is analyzed. The dynamic pressure contact distribution pattern is set, and the variation of the fluid film thickness at the interface is predicted. The dynamic pressure applied to the interface texture contact and the equivalent uniformized microelement load balance equation between the tooth surfaces are updated in real time to achieve the minimum thickness index of the dynamic pressure fluid film.

This study validates the dynamic pressure contact load-bearing characteristics of textured meshing interfaces under TEHL through a pin-slider-disc simulation bench test, analyzes the equivalent homogenization fluid film load-bearing performance of the microelementary textured interfaces within the meshing domain, and evaluates the dynamic pressure fluid film load-bearing performance enhancement and efficiency-shared thermoelastic lubrication performance of the meshing interface microelementary textures, optimizing its prediction model.

## 3. Dynamic Pressure Contact Simulation Experiment of Textured Microelement Meshing Interface

### 3.1. Biological Microprocessing Removal Mechanism

**Figure 8:**
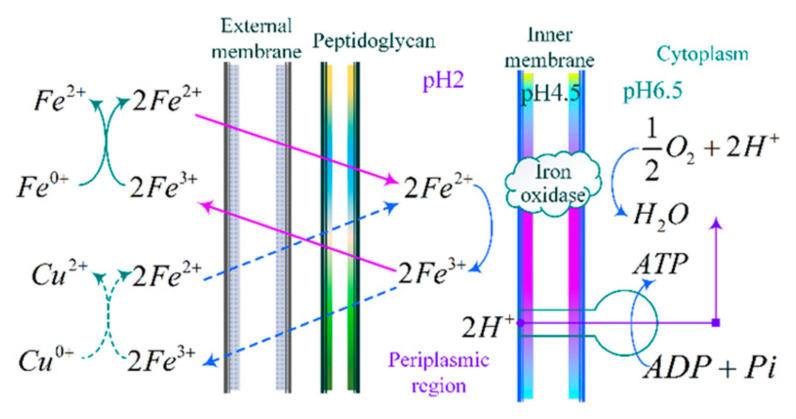
Biological microprocessing removal mechanism of Acidithiobacillus ferrooxidans.

Biological microprocessing is a new fabrication method that emerged using physics and chemistry, i.e., bio-removal, a manufacturing method that utilizes microorganisms as preparation tools to process and remove materials (etching materials). Microorganisms synthesize organisms for the material enrichment, leaching, or etching of solid materials based on material processing mechanisms that take in the nutrients needed to sustain their lives and the energy needed to grow and reproduce [39,40,41]. The energy required by microbe-specific bacilli (e.g., ferrous oxide and sulfur oxide) for their own growth, on the other hand, utilizes the chemical energy released during the oxidation of metals such as Cu, Fe, Al, and Cr (monobasic) and recognizes carbon dioxide as a carbon source to support their own nourishment and reproduction, as shown in Figure 8. Currently, research results have been achieved in the field of biomicrofabrication in the international arena, and a variety of methods have emerged, such as bioplastic processing, deposition processing, and microfabrication removal. In particular, biomicrofabrication removal is highly valued in the manufacturing industry due to its unique preparation method and non-polluting and environmentally friendly advantages. Similar to biometallurgy, which focuses on the direct and indirect oxidative coexistence mechanism revealed by Thiobacillus ferrooxidans, biomicrofabrication removal has gained momentum, especially over the last two decades and has attracted significant attention.

#### 3.1.1. Indirect Oxidation Mechanism

Ferrous oxide thiobacillus cytosol consists of an inner membrane, an external membrane, a periplasmic region, and a peptidoglycan; iron oxidase is stored in the inner membrane and periplasmic region, and the bacillus culture fluid is stored across the cytosol to the periplasmic region. Under the catalytic action, iron oxidase will be converted (loss of 1 electron) to (a metal etching agent commonly used in industry), and will provide the electrons through the proteins, and a series of enzymes will provide the oxygen molecules transferred to the receptor to take up in the bacteriophage cells and produce energy release.

The energy can be supplied to the bacillus to reproduce and grow and promote its intracellular ADP conversion to ATP, which, through the bacillus cell membrane, will be converted to $Fe^{3+}$; discharge outside the organism, $Fe^{3+}$ can be oxidized and achieve the removal of Cu, Fe, Al, Cr, and other monobasic metals, and can also be reduced to $Fe^{2+}$ and then oxidized into $Fe^{3+}$ by the bacillus, and then the cycle begins again. The chemical reaction formula is as follows [42]:

$$\begin{array}{l} 2Fe^{2+}+\frac{1}{2}O_{2}+2H^{+}\overset{AcidiThiobacillus\;ferrooxidans}{\to }2Fe^{3+}+H_{2}O\\ 2Fe^{3+}+J^{0}\to 2Fe^{2+}+J^{2+} \end{array}$$

where J denotes single matrix metals such as Cu, Fe, Al, and Cr.

#### 3.1.2. Direct Oxidation Mechanism

Based on the aforementioned mechanism of metal removal by the microprocessing of Thiobacillus ferrooxidans, Thiobacillus ferrooxidans do not apply metal directly, but convert $Fe^{2+}$ to $Fe^{3+}$ by oxidation and then oxidize the substrate metal in an indirect process, which is described as an indirect oxidation mechanism. The direct oxidation mechanism is revealed in depth. It is observed that Thiobacillus ferrooxidans can directly touch the microfabricated materials through the adsorption of extracellular polymers, and the enzymes produced by the metabolism of the bacterium are delivered to the interface of the microfabricated parts by the extracellular polymers, directly oxidizing the metal materials to realize the interfacial micro-removal treatment process.

#### 3.1.3. Experimental Sample Preparation

**Figure 9:**
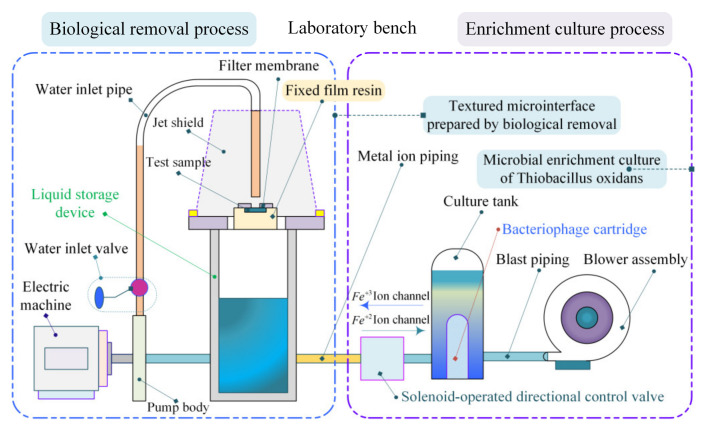
Microfabrication MTME experimental procedure for Thiobacillus ferrooxidans.

Considering the above mechanism of metal oxidation removal by Thiobacillus microfabrication, an interfacial texturing microelement scale is prepared, whose experimental benches are shown in Figure 9. In turn, crater groove microelements, with specified external morphology dimensions and row densities, are constructed.

**Figure 10:**
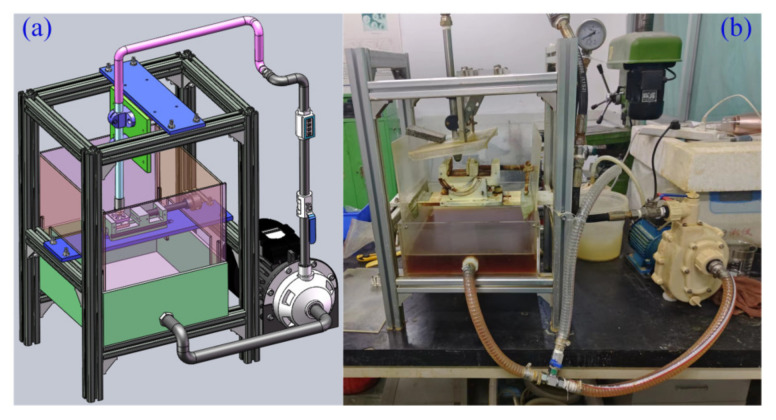
Model and object of experimental platforms for biomicrofabricated MTMEs: (**a**) experimental platform model; (**b**) experimental platform object.

**Figure 11:**
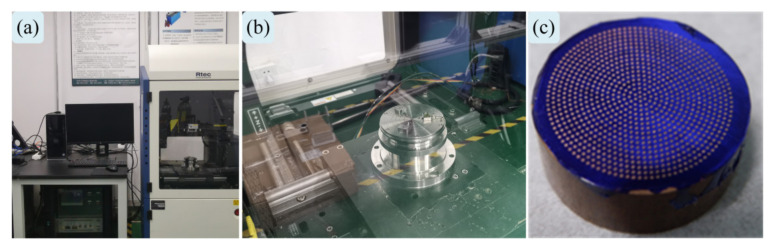
Friction behavior and wear properties of textured microelement interfaces test rig: (**a**) MFT-5000-RETC testing machine; (**b**) specimen fixture; (**c**) textured microelement sample.

Biomicromachining removes the texturing microelement experimental platform model, along with its physical objects, as shown in Figure 10. The pin-slider-disk type simulation experiment is based on the friction behavior and wear performance test platform (MFT-5000, RETC, Fremont, CA, USA), considering the actual friction conditions in the contact domain, integrating the multi-technology analysis capability, transmitting the multi-detection signal function, and realizing the modular customized experimental design (e.g., reciprocating high-speed sliding, pin rotating disk, etc.). The texturing microelement interface friction behavior wear performance test bench is shown in Figure 11.

**Figure 12:**
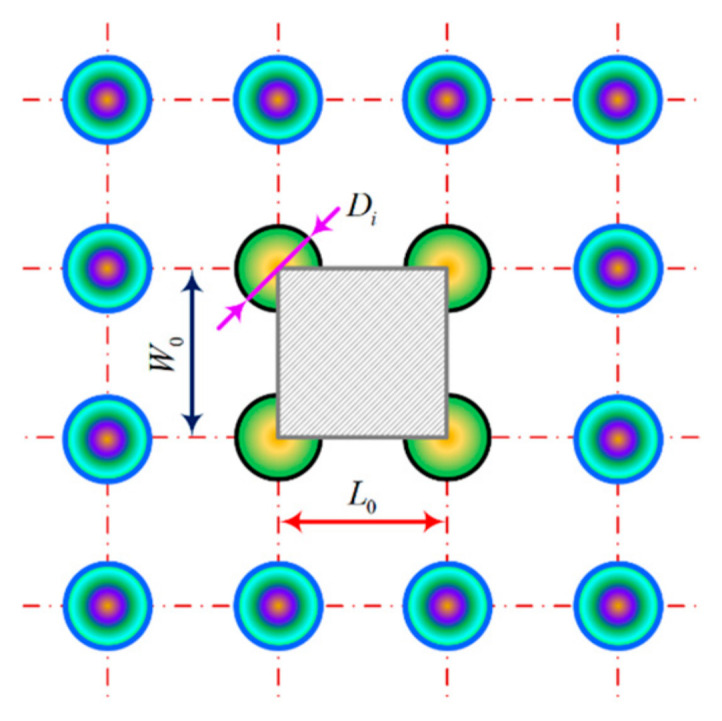
Solution model for coverage ratio of MTME interface in microprocessing.

Interfacial textured microelement preparation should follow the configuration of the morphology and scale characterization to meet the design indicators; in particular, the textured microelement occupies the boundary surface area coverage ratio, i.e., the microprocessing textured microelement interface coverage ratio numerical solution model, as shown in Figure 12, which avoids sharing the meshing interface maximum stress. The equivalent texture unit contact volume determines the real area of the multiscale interface meshing domain, confirming that the non-arbitrary characterization of the MIMT microelement scale configuration and the actual area of the microelement peak body are taken as the evaluation basis for meshing ASLBC between the steady-state TEHL line contact interfaces.

Setting the diameter of a single microelement of the texture as $D_{i}$, $W_{0}$ and $L_{0}$ represent the longitudinal and transverse distances between neighboring domains of the weave microelements, respectively. The texturing elements interface coverage ratio $R_{i}$ is solved by the following equation [43]:

(12) $$R_{i}=\frac{\pi D_{i}^{2}}{4W_{0}L_{0}}\times 100\%$$

### 3.2. Texturing Microelement Interface Model Simulation Experiment

**Figure 13:**
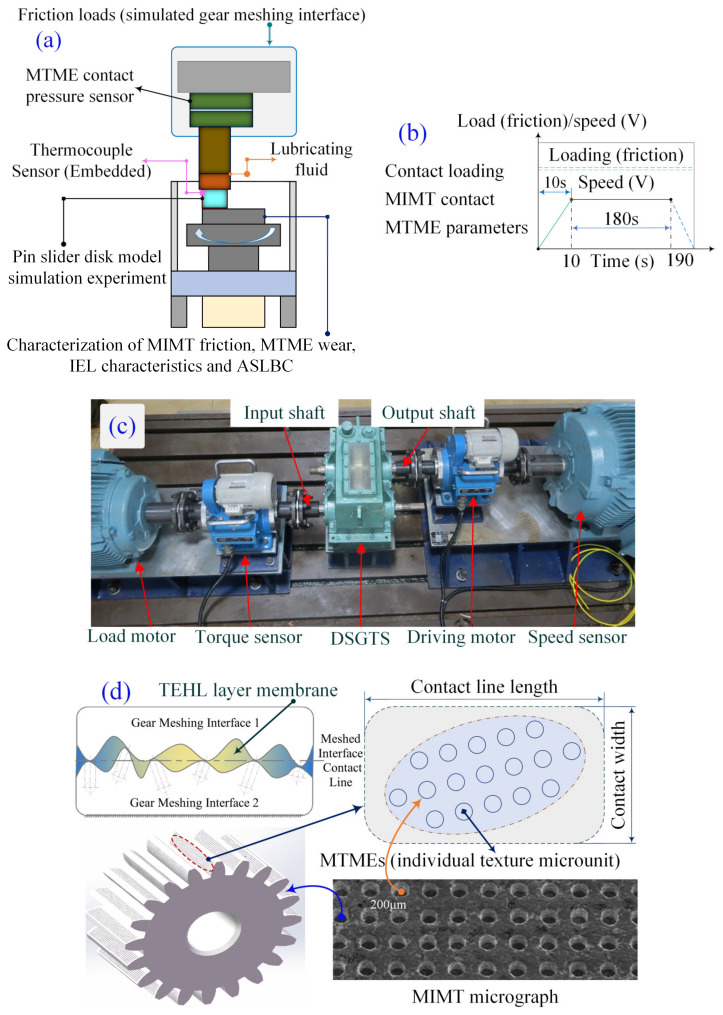

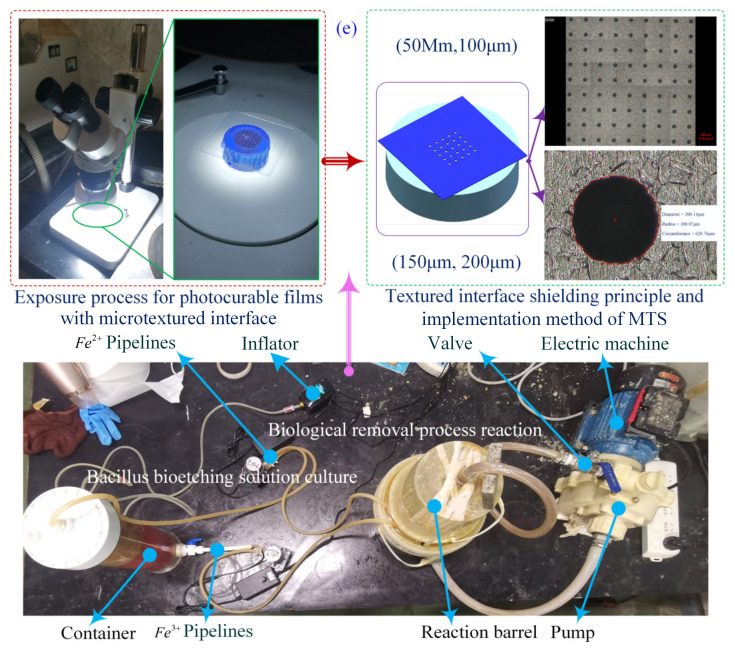
Detailed description of the thermoelastic dynamic contact simulation experiments: (**a**) simulation test rig for line contact of pin-block disk; (**b**) experimental validation of simulation analysis; (**c**) test rig for actual texturing of meshing interface contacts; (**d**) TEHL dynamic pressure interface temperature measurement; (**e**) preparation of microtextured samples by biological microfabrication removal method.

A pin-slider disk-type experimental platform is proposed for simulating and verifying the equivalent homogenized interfacial load-bearing performance of the TEHL under the influence of the correlation of multiscale texturing microelement parameters. The textured interface microelement dynamic pressure contact simulation experiment is based on a pin-slider disk-type platform, and the meshed tooth surface line contact roll–slip model counts the microelement parameter scale characteristics, and these microelement parameters present a periodic homogeneous arrangement within the mesh line region rather than parallel to the textured interface morphology along the roll–slip direction in the specified mesh line domain (see Figure 13a–c). In the experimental simulation verification, the pin diameter is set to ϕ25mm, and its material is 40Cr, and the functional disk surface (ϕ19.5±0.3mm) is achieved by grinding. To explore the mechanism of biological micromachining the metal single substrate and its preparation efficiency, and to structure the contact area of the disk surface microelement morphology, the depth of MTME is preset to be 30 μm; its widths are 50 μm, 100 μm, and 200 μm; and the rate of coverage of the disk surface area can be up to 80%. Mechanical and electronic information fusion sensing devices (multisource sensors) are used to collect real-time simulated meshing load and interface friction contact behavior data. The lead port is located at 0.50 mm below the center of the disc functional surface, the thermocouple sensor built into the MTME continuously monitors the thermoelastic fluid dynamic contact temperature, the applied load is set to 100 N and 200 N, and the speed values of the slip line contact simulation experiments are 1.0 m/s and 1.0 m/s and 200 N, respectively. The applied loads are set to 100 N and 200 N, the sliding line contact simulation experimental speeds are 1.0 m/s and 2.0 m/s, the meshing period is controlled within 180 s (the entire cycle time), and the thermoelastic fluid is continuously sent from the input end to the pin-slider-disk texturing area interface domain (simulating the contact area meshing domain of the gear subcontact area) (see Figure 13d,e).

**Figure 14:**
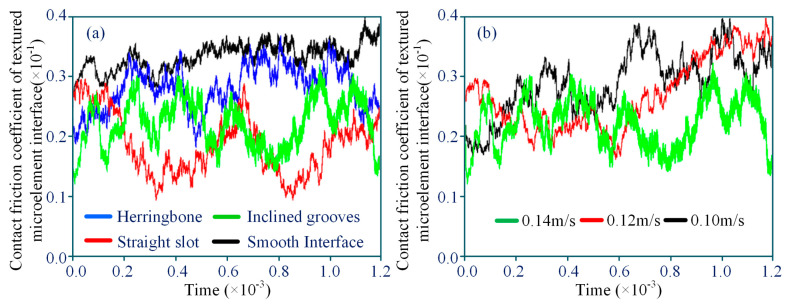
Time-varying characteristics of contact friction at interfaces with different textured m-croelements: (**a**) friction coefficient curves of textured interfaces; (**b**) friction coefficient curves of smooth interfaces without microelements at different linear velocities.

The time-varying contact friction characteristics of different textured microelement interfaces are analyzed. The time-varying curves of the textured interface friction coefficients are shown in Figure 14a. The blue curve represents the time-varying law of the contact friction coefficient of the interface of the microelement herringbone configuration, the green curve represents the time-varying law of the contact friction coefficient of the interface of the microelement inclined groove configuration, and the red curve represents the time-varying law of the contact friction coefficient of the interface of the microelement straight slot configuration. The black curve represents the time-varying law of the contact friction coefficient of the smooth interface, without microelement configuration. The time-varying friction coefficients of the microelement interfaces of the above four types of textured interfaces, from the initial friction to the stable contact friction stage, show that the interface contact friction coefficients of the interfaces with textured microelements are much smaller than those of the smooth interfaces with no-microelement configurations, with the interfacial contact linear velocity of 0.10 m/s and the load set at 10 N. The friction characteristics are much better than those of the interfaces without microelement configurations, which exhibit better friction time-varying characteristics. The friction coefficient of the microelement straight slot interface is the smallest, followed by that of the microelement diagonal groove interface, and then that of the microelement herringbone interface. A comparison of the time-varying trend of the friction coefficient of the smooth interface, without microelements, at different linear velocities is shown in Figure 14b, which shows that the friction coefficient decreases significantly when the interface contact linear velocity increases.

**Figure 15:**
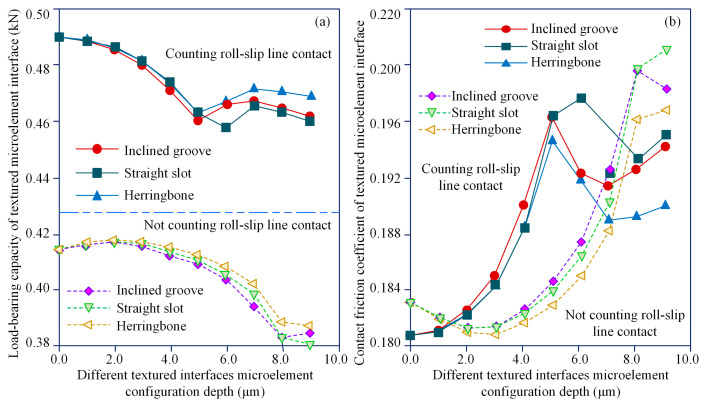
Lubrication and load-bearing properties of the textured interface in different MTME configurations: (**a**) time-varying load-bearing capacity of textured interface versus MTME depth; (**b**) time-varying contact friction coefficient of the textured interface versus MTME depth.

An analysis of the lubrication characteristics of the textured interface under different microelement configuration parameters, as shown in Figure 15, reveals that the microelment depth affects the load-bearing performance of the texture interface in a regular manner, and that the load-bearing capacity of the microelement interface under the co-ndition of roll–slip TEHL is not less than that of the unaccounted-for example. The micro-element herringbone configuration texturing interface displays the best load-bearing c-apacity, followed by the microelement diagonal groove configuration interface, and then the microelement straight slot configuration interface. This phenomenon can be reasonably explained by the fact that the herringbone configuration microelement interface exerts a more significant step effect compared to those of the other two microelement configuration interfaces. When roll–slip is not considered, the load-bearing capacity of the microelement interface and the contact friction coefficient curves of the tooth surface of the te-xturing configuration flatten out, as shown in Figure 15a,b. When roll–slip is considered and the microelement depth is increased, the load-bearing capacity of the textured inter-face decreases instead of increases. When the microelement depth is set to 5.0 µm, the herringbone configuration textured interface load-bearing capacity decreases to its lowest level. The herringbone configuration textured interface load-bearing capacity varies slowly with increasing microelement depth. Compared to the case where roll–slip is not accounted for, the textured interface load-bearing capacity decreases with increasing microelement depth. The reason for this that the increase in microelement depth tends to enhance the axial step load-bearing effect of the textured interface, while weakening hydrodynamic pressure effect in the circumferential direction.

## 4. Load-Bearing Experiments of Lubricated Meshing Interfaces of Textured Microelements

### 4.1. Meshing Interface Lubrication Load-Bearing Test Bench

**Figure 16:**
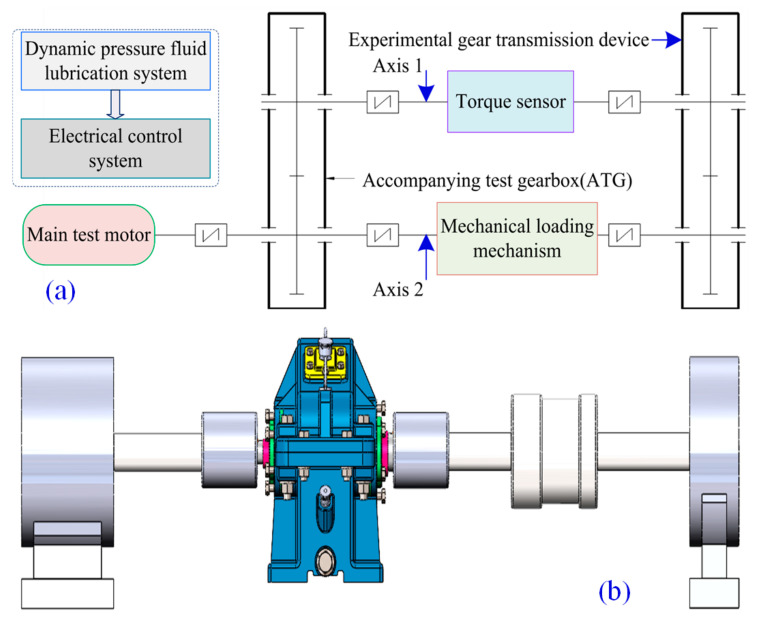

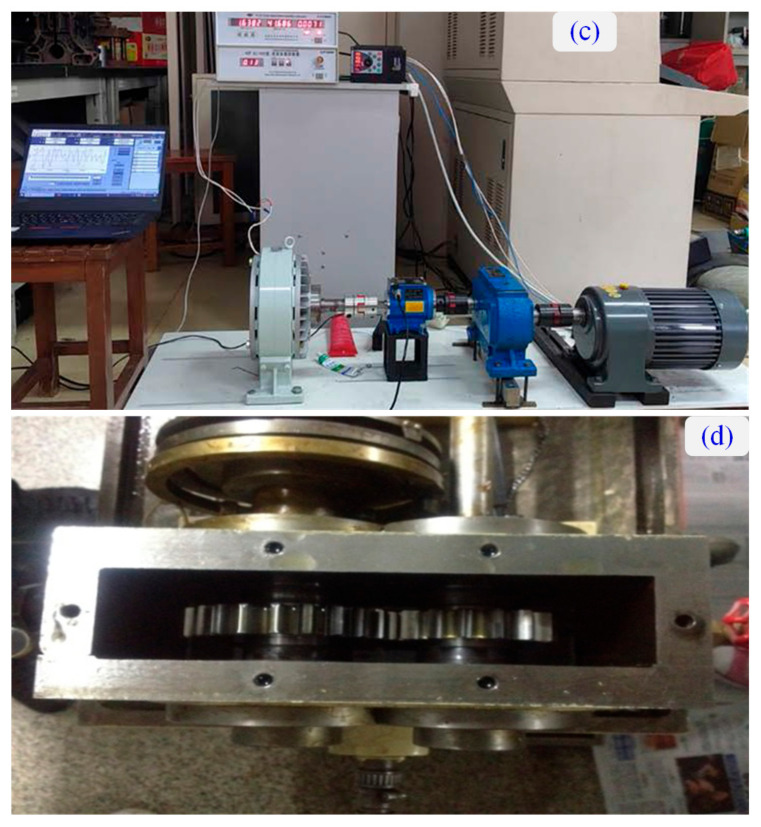
Textured microelement lubrication interface load-bearing test bench: (**a**) layout sketch; (**b**) three-dimensional drawing; (**c**) type platform; (**d**) experimental gear unit.

The meshing interface lubrication load-bearing experiment to power a non-open platform layout is shown in Figure 16. The use of this setup means that the difference between the dynamic and static friction torque can be ignored, and the torque can be precisely controlled by adjusting the excitation current, effectively regulating the reverse load value. The gear transmission device is forced-lubricated using thin oil, the dynamic pressure fluid is CD40, and its kinematic viscosity is 14.31 mm^2^/s (100 °C). The technical parameters of the gear transmission device of the meshing interface lubrication load-bearing test bench are shown in Table 2. The power time-varying characteristics of the gear unit are monitored, and the transient load is recorded by the torque sensor, enabling the evaluation of the lubrication power output stability of the meshing interface.

### 4.2. Characterization of Textured Interface Meshing Load-Bearing Behavior

**Figure 17:**
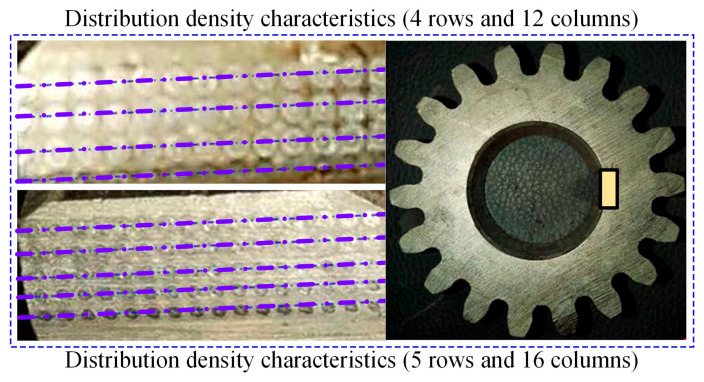
Characterization of textured interface meshing loads at different distribution densities.

The textured interface meshing load-bearing behavior experiment is divided into three verification groups: untextured meshing interface, cloth state density characteristics of the large and sparse micrometamorphic interface, and cloth state density characteristics of small and dense micrometamorphic interface, considering the same working conditions and experiments. The main motor speed is set to 1500 rpm, the meshing pair runs continuously 10^5^ times to stop the contact, and the experimental time of the meshing load-bearing behavior of a single group of micrometamorphic surfaces is not less than 4 h. After the completion of the experiments of a single working condition, the contacting vice (gear pair) is replaced, and the supporting bearings and seals are checked, ensuring that they are ready for the next experimental validation of the working condition. As shown in Figure 17, based on Thiobacillus microfabrication to remove the metal oxidation mechanism, multiscale textured micrometamorphic meshing interfaces are produced on the active tooth surface, in which large and sparse micrometamorphic interfaces (cloth state featured with 4 rows and 12 columns), as well as small and dense micrometamorphic interfaces (cloth state featured with 5 rows and 16 columns), are biologically processed.

In order to investigate the meshing interface anti-scuffing load-bearing capacity (i.e., the contact effect of anti-thermoelastic scuffing fatigue damage) under the condition of MTME lubrication, the characterization of meshing load-bearing capacity focuses on the morphological characteristics of the thermoelastic fatigue damage microelement of the meshing tooth surface when the texture interface exhibits anti-scuffing failure, as well as the friction contact power consumption and the smoothness of the transmission transient meshing load-bearing torque and other parameters. The experimental behavior of the meshing load-bearing performance of the texture interface is characterized by the instantaneous characteristics of the contact transmission of the micro-lubrication interface and the texture interface anti-thermoelastic scuffing failure. The transient characterization of the micro-lubrication interface contact transmission indicates that the torque sensing element (sensor) records the meshing load-bearing torque data of the texture interface in real time, compares the smoothness of the meshing load-bearing torque, and calculates the friction contact consumption of the meshing pair by considering the transmission power.

The characterization of the thermoelastic scuffing failure of the textured interface meshing load is described as the characterization of the textured tooth surface and the cross-section of the meshing gear body. The single gear teeth are cut along the root area using a wire cutter, washed and dried by ultrasonic wave technology in an alcohol medium, and the failure state of the anti-thermoelastic scuffing damage of the texture interface meshing load is analyzed and characterized using a CCD microscope (G-300C-Gaopin, Shenzhen, China). The 12 mm × 6 mm × 6 mm texture element is cut from the pitch circle of the meshing interface, cleaned and dried, inlaid with resin, ground, and polished. The thermoelastic deformation and anti-scuffing damage are scanned by an electron microscope (SEM) (S-3400N-Hitachi, Tokyo, Japan) along the depth direction of the interfacial microelement via etching and polishing with a 5% nitric acid alcohol solution.

## 5. Experimental Simulation and Analysis of Lubricated Microelementary Meshing Load-Bearing of Textured Interface

### 5.1. Experimental Simulation of Meshing Load-Bearing Torque Stability

Statistically based experimental simulation of the meshing load-bearing output torque at the textured interface is used to obtain the root mean square value of the meshing load-bearing torque (with a set range of 1.0 min) and the value of the meshing load-bearing variance.

**Figure 18:**
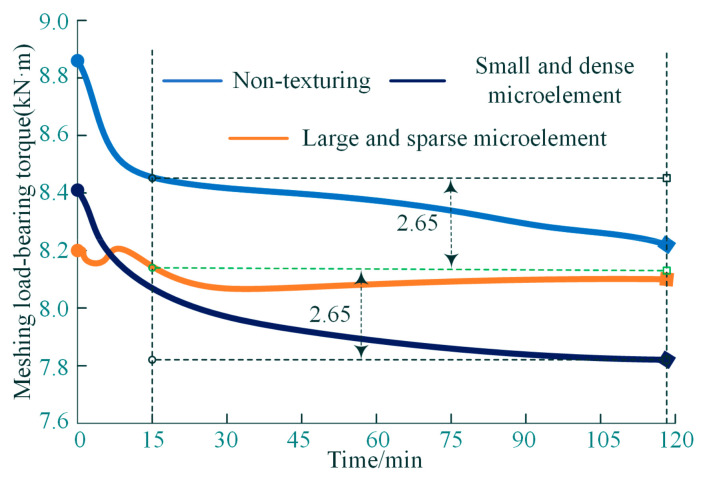
Time-varying curves of output torque for meshing loads.

In Figure 18, the experimental process of textured microelement interface type simulation can be divided into two stages: the initial loading (0–15 min) and the steady meshing state (15–120 min). During the initial period, the output load torque of the meshing pair showed a significantly decreasing trend. During the steady meshing state, the variation relationship between the output load torque and the input power is consistent, and the variance (fluctuation) of the torque of the meshing load is only ±2.65%.

**Figure 19:**
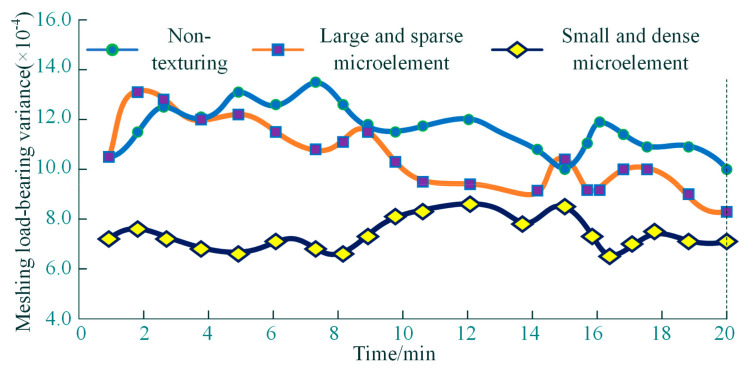
Time-varying curves of output torque variance for meshed loads.

In Figure 19, the load-bearing steady-state performance of the output torque during the initial contact history of the texturing interface is analyzed, based on the meshing load output torque variance. The analysis shows that small and dense microelements outperform large and sparse microelement texturing features during the steady-state torque output of the meshing load, while large and sparse microelement texturing features outperform the interface with no texturing features, which further confirms that the microelement texturing meshing interface has a greater impact on the stable torque-bearing capacity of the output torque, and that microtexture units with small and dense texturing features are more conducive to the load-bearing stable operation of the meshing interface.

### 5.2. Microelemental Morphometric Analysis of Meshing Interface Anti-Scuffing Failures

**Figure 20:**
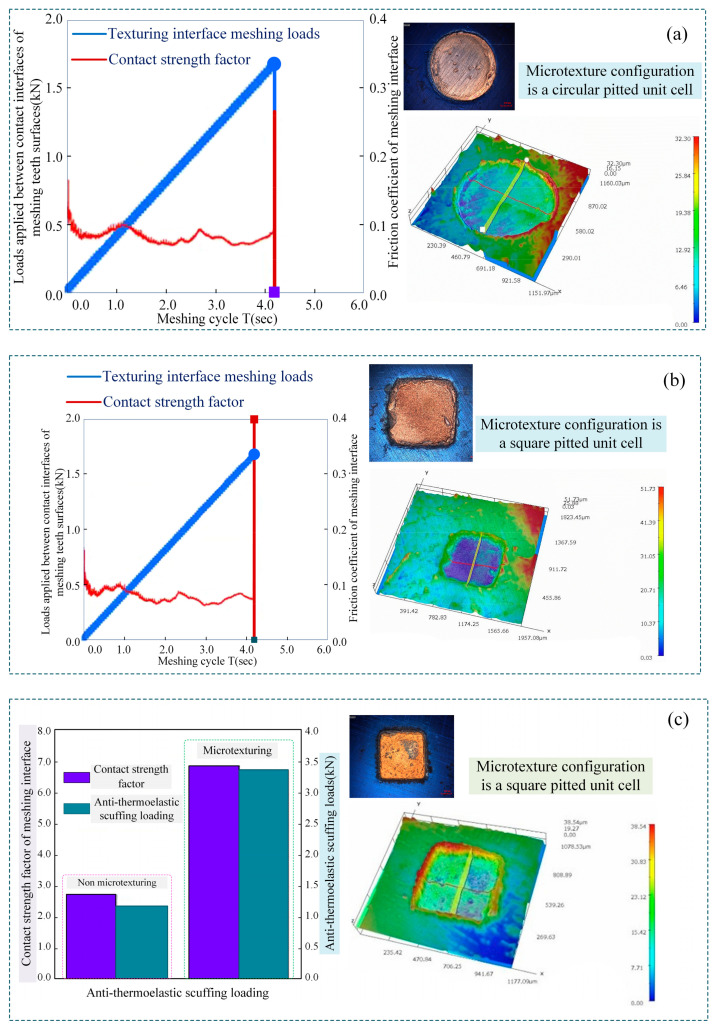
The morphological characteristics of meshing interface anti-scuffing failures of different microelement configurations: (**a**) changing law of friction coefficient and meshing load-bearing of circular pits microtextured interface; (**b**) changing law of friction coefficient and meshing load-bearing of square pits microtextured interface; (**c**) ASLBC and contact strength factor for microtextured and non-microtextured meshing interfaces.

In the process of incurring meshing interface anti-scuffing failures, there is a competition between tooth surface lubrication and microelement load-bearing. Discrete microtexturing unit configurations are introduced, the real-time changes of the aforementioned phenomena are induced under the same working conditions, and the morphological characteristics of the meshing interface anti-scuffing failures of different microelement configurations are analyzed, as shown in Figure 20.

Figure 20a shows the variation law of load-bearing between the meshing interface friction characteristics (friction coefficient) of the microtexture meshing interface of the circular concave element and the contact interface of the meshing gear. The results show that with the increase in applied loads between the meshing interfaces, the dynamic pressure fluid is introduced into the tooth surface contact area from the circular element pits, and the dynamic pressure lift effect of the fluid is generated at the pits. For TEHL lubrication, the load-bearing mechanism of the microelement body changes into the rheological model of the meshing interface under mechanical contact dynamic pressure. Under the applied loads effect, the fluid is squeezed out of the contact-bearing domain and generates the dynamic fluid pressure, which shares part of the normal loads between the interfaces.

Figure 20b shows the variation law of the load-bearing between the meshing interface friction characteristics (friction coefficient) of the microtexture meshing interface of the square concave element and the contact interface of the meshing gear table. It is observed from the figure that the presence of the textured microelement can cause the dielectric film between the gear pair meshing interface to generate dynamic pressure under hydrodynamic thermoelastic lubrication, which is due to the formation of a non-divergent film wedge along the velocity direction of the dielectric layer between the edge of the square micro-concave element and the meshing interface of the gear pair. The multi-objective optimization of the size and shape of the convergent film wedge depends on the distribution characteristics of the micro-concave elements at the texture interface, generating the bearing dynamic pressure in the liquid membrane between the textured interface. The liquid layer film pressure at the right edge of the square micro-concave unit continues to rise until the maximum film pressure peak is reached, which is attributed to the convergent film wedge formed between the right edge of the micro-concave unit and the meshing interface of the gear pair. The square pitted element is more likely than the round pitted element to generate dynamic loads on the liquid layer membrane, thus enhancing textural loads performance of the meshing interface. The pressure of the liquid layer membrane at the left edge of the pitted element shows a decreasing trend, which is due to the existence of a non-convergent membrane wedge.

Figure 20c shows the time-varying trend of the ASLBC of the meshing interface, with and without microtexture, along with the contact strength factor. The analysis shows that the meshing bearing contact strength factor of the microtextured interface is not only a descriptive function of the meshing loads when the thermoelastic scuffing fails but also an expressive function of the friction coefficient (the rolling slip velocity is assumed to be constant in the experimental test).

**Figure 21:**
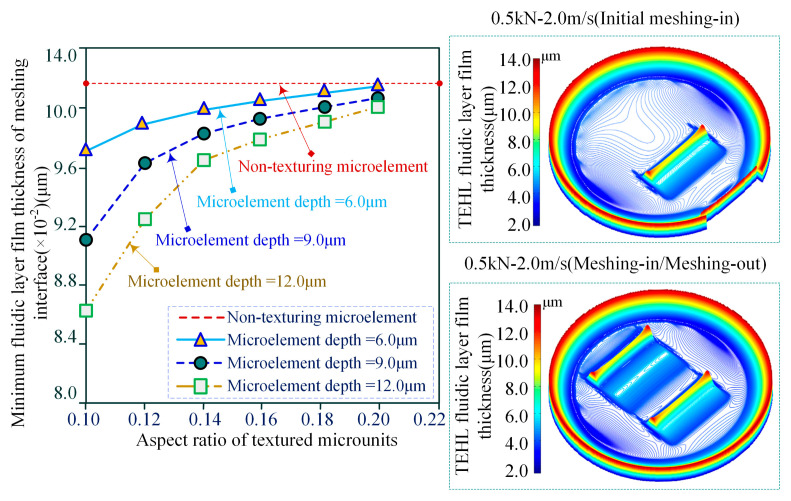
Time-varying of TEHL minimum homogeneous layer film thickness for the multiscale textured interface of micro-concave pit.

Considering that the load borne by the meshing interface microtexture is set to 0.5 kN, and the slip speed is 2.0 m/s, the time-varying law of the minimum homogeneous layer film thickness of the multiscale characteristic texture interface with micro-pits is analyzed under TEHL conditions, as shown in Figure 21. The simulation results show that the sliding contact linear velocity is 2.0 m/s when the textured interface is subjected to a load of 0.5 kN. The effect of element depth (configurational scale) on the contact pressure of the meshing load and the thickness of the thermoelastic homogeneous layer is revealed. The time-varying characteristics of meshing contact interface pressure and homogeneous layer film thickness under different microelement depths and aspect ratios are compared and analyzed, in which the depths of the three microelement configurations are 6.0 μm, 9.0 μm, and 12.0 μm, respectively, and the aspect ratios of the texturing microelements are set to be in the range of 0.10–0.20. The core objective of MIMT is to enhance the contact load-bearing capacity of the meshing interface, and the thickness of the thermoelastic homogeneous layer film with microtexture unit characteristics should be greater than that of the non-microtexture interface, which in turn extends the pressure distribution of the meshing interface to a larger contact area. The discrete contact interface pressure at the front edge of the mesh element boundary formed two peaks of microelements. When the aspect ratio of the meshing interface MTME is not large enough, the contact area is wide, the interface load is stable in the meshing domain, and the peak value of the discrete contact interface pressure microelements at the back edge of the texture boundary is relatively high, achieving the load balance of the meshing interface microelements. This high discrete contact interface pressure microelement peak results in a thermoelastic homogeneous film thickness that is even smaller than that of the minimum homogeneous film with an unstructured meshing interface under the same conditions.

**Figure 22:**
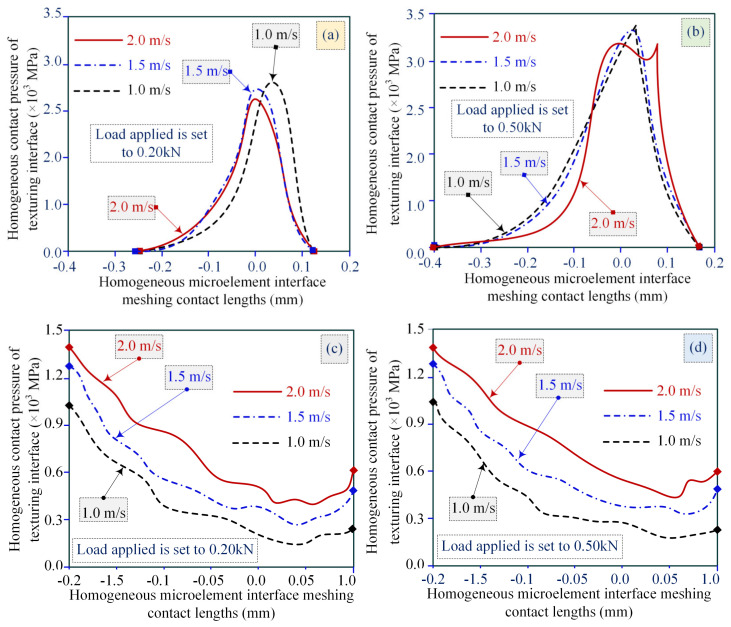
Time-varying laws of meshing interface homogeneous microelement contact pressure with different sliding line speeds. (**a**) Load applied set to 0.2 kN (with MIMT); (**b**) Load applied set to 0.5 kN (with MIMT); (**c**) Load applied set to 0.2 kN (without MIMT); (**d**) Load applied set to 0.5 kN (without MIMT).

As shown in Figure 22, the loads borne by the meshing interface are set to 0.2 kN and 0.5 kN, and the slip contact linear velocities are set to 1.0 m/s, 1.5 m/s, and 2.0 m/s, which are analytically accounted for by the changing law of homogenized microelement contact pressure at the meshing interface under different slip linear velocities. Based on the simulation of the lubrication performance and the friction/wear behavior of the texturing microelement, the ASLBC capability focuses on the microelement homogenized meshing interface of the slip line contact, illustrating the time-varying effect of the homogenized microelement interface pressure related to the slip contact linear velocity via numerical calculation and the simulation model, indicating that the slip linear velocity constrains the formation of the thermoelastic homogeneous layer film and the improvement of the meshing bearing capacity of the microelement interface.

Considering the line contact interface microelement characteristics, a thinner thermoelastic homogeneous layer film can weaken the microelement interface meshing load-bearing capacity. Increasing the line contact slip velocity of the meshing interface will make the microelement multiscale characteristics superior in terms of the thermoelastic homogeneous layer thickness and the meshing load-bearing capacity of the texturing interface. It is determined that increasing the slip line velocity leads to the formation of a thicker thermoelastic homogeneous layer film, which in turn constrains the texturing interface meshing pressure distribution and the microelement peak body contact amplitude. The texture microunit properties are characterized by two peaks of meshing pressure contact at the edge of the configuration and a flat region with a lower contact pressure in the middle domain of the meshing region.

The decreased amplitude of the thermoelastic homogeneous layer thickness corresponding to the contact peak of the meshing pressure may be even less than the minimum homogeneous layer thickness, based on the absence of the interface texture. A validated and feasible analytical method consists of embedding the discrete contact analysis method into the texturing interface to form a thermoelastic homogeneous layer of the meshing tooth surface. In addition, the minimum homogeneous layer thickness appears near the contact front of the textured microelement boundary.

In summary, a dynamic pressure model of homogeneous flow at the textured meshing interface under thermostatic lubrication is established, geometric configuration parameters with multiscale characteristics of the textured meshing interface are proposed, the action mechanism of the textured interface elements in thermoelastic lubrication is discussed, and the correlation law between the textured interface lubrication performance and the meshing load-bearing capacity is revealed. The effect of optimization on the meshing bearing capacity is clarified, and it is determined that the contact velocity of the slip line is obviously related to the meshing load-bearing capacity of the microtextured interface.

## 6. Conclusions

With the increasing requirements of modern high-end deep-sea equipment for heavy-duty capacity, high power density, long service life, and high reliability, the TEHL characteristics of meshing load-bearing performance gears has become a critical issue for power conversion devices and/or various transmission systems. Especially in the deep-sea space environment, the stability of the DSGTS is extremely important. Considering the multiscale configuration parameter feature analysis method, a microelement homogeneous flow pressure contact model is established, and the meshing ASLBC induced by the thermoelastic effect of MIMT discrete element microflow lubrication is introduced to induce the meshing ASLBC of the texturing interface discrete unit, and a microelementary contact meshing bearing model is proposed for the texturing interface homogeneous TEHL, which reveals the characteristics of the TEHL dynamic pressure synergistic effect among MTME interfaces and the meshing bearing capacity related to anti-scuffing ability. The correlation mechanism between the texturing interface homogeneous TEHL, the multiscale friction behavior, and the MTME dynamic pressure contact load-bearing is elaborated, and a numerical prediction analysis model is pro-posed, which is used to simulate the MTME lubrication with multiscale non-macroscopic features and to evaluate the anti-thermoelastic scuffing of DSGTS meshing load-bearing. Based on the theoretical study and experimental analysis results, the following conclusions are drawn:

(1) The output loading torque of the textured interface meshing pair shows a significant decreasing trend in the meshing-in period, the relationship between the output load torque and the input power of the textured interface meshing pair is consistent during the thermal steady-state meshing process, and the meshing load torque fluctuation rate (i.e., variance) is controlled within ±2.65%. This process is regarded as a bridge connecting the homogeneous IEL characteristics of microscopic dynamic pressure discrete MTMEs with the ASLBC of MIMTs.
(2) The proposed MIMT contact model theoretically predicts the evolutionary time-varying characteristics of the MTME thermoelastic lubrication behavior of the textured dynamic pressure interface and demonstrates that the MTME geometric scale is the most influential configuration parameter for the homogeneous microflow pressure laminar film load-bearing problem between the contacting meshing teeth surfaces.
(3) The friction coefficient of the dynamic pressure contact interface with the microelements decreases by 14% to 26%, which in turn verifies the correlation between the thermoelastic flow homogenization lubrication behavior of the MIMT and the meshing interface load-bearing contact mechanism, which provides a new way to further study the IEL performance and the ASLBC of the textured interface under the coupling of the MTMEs characteristic parameters.

The research confirmed that the meshing interface microtexture (MIMT) applied on the TEHL tooth surface of a deep-sea gear transmission system (DSGTS) is prone to improve the homogeneous interface enriched lubrication (IEL) characteristics, especially for the deep-sea space environmental operating conditions of time-varying high torque. The above findings have important implications for further in-depth study regarding microtextured gear design and meshing IEL and dynamic anti-scuffing load-bearing capacity (ASLBC) optimization synergy.

## Figures and Tables

**Table 1:** Defining gear geometry parameters of finite element model analysis.

Geometric Parameter	Driving Gear	Driven Gear
Tooth number	42	51
Modulus (mm)	3	3
Pressure angle (°)	20	20
Tooth width (mm)	60	60
Poisson’s ratio	0.26	0.26
Elastic modulus (GPa)	210	210
MTME(μm)	20–200	20–200
Lubricating oil	CD40	CD40

**Table 2:** Meshing interface lubrication load-bearing test bench gear device technical parameters.

Experiment Names	Technical Parameters
Rated power	3.0 kW
Loading speed	0–3000 rpm
Load torque	0–500 N·m
Running time	To be defined

## Data Availability

The original contributions presented in this study are included in the article. Further inquiries can be directed to the corresponding authors.
